# Anti-GBM Glomerulonephritis Involves IL-1 but Is Independent of NLRP3/ASC Inflammasome-Mediated Activation of Caspase-1

**DOI:** 10.1371/journal.pone.0026778

**Published:** 2011-10-27

**Authors:** Julia Lichtnekert, Onkar P. Kulkarni, Shrikant R. Mulay, Khader Valli Rupanagudi, Mi Ryu, Ramanjaneyulu Allam, Volker Vielhauer, Dan Muruve, Maja T. Lindenmeyer, Clemens D. Cohen, Hans-Joachim Anders

**Affiliations:** 1 Nephrological Center, Medical Policlinic, University of Munich, Munich, Germany; 2 Division of Nephrology and Hypertension, Department of Medicine, Department of Medicine and the Immunology Research Group, Institute of Infection, Immunity and Inflammation, University of Calgary, Calgary, Canada; 3 Division of Nephrology and Institute of Physiology, University Hospital and University of Zurich, Zurich, Switzerland; Institut National de la Santé et de la Recherche Médicale, France

## Abstract

IL-1β and IL-18 are proinflammatory cytokines that contribute to renal immune complex disease, but whether IL-1β and IL-18 are mediators of intrinsic glomerular inflammation is unknown. In contrast to other cytokines the secretion of IL-1β and IL-18 requires a second stimulus that activates the inflammasome-ASC-caspase-1 pathway to cleave pro-IL-1β and -IL-18 into their mature and secretable forms. As the NLRP3 inflammasome and caspase-1 were shown to contribute to postischemic and postobstructive tubulointerstitial inflammation, we hypothesized a similar role for NLRP3, ASC, and caspase-1 in glomerular immunopathology. This concept was supported by the finding that lack of IL-1R1 reduced antiserum-induced focal segmental necrosis, crescent formation, and tubular atrophy when compared to wildtype mice. Lack of IL-18 reduced tubular atrophy only. However, NLRP3-, ASC- or caspase-1-deficiency had no significant effect on renal histopathology or proteinuria of serum nephritis. *In vitro* studies with mouse glomeruli or mesangial cells, glomerular endothelial cells, and podocytes did not reveal any pro-IL-1β induction upon LPS stimulation and no caspase-1 activation after an additional exposure to the NLRP3 agonist ATP. Only renal dendritic cells, which reside mainly in the tubulointerstitium, expressed pro-IL-1β and were able to activate the NLRP3-caspase-1 axis and secrete mature IL-1β. Together, the NLRP3-ASC-caspase-1 axis does not contribute to intrinsic glomerular inflammation via glomerular parenchymal cells as these cannot produce IL-1β during sterile inflammation.

## Introduction

The induction of proinflammatory cytokines is a hallmark of renal inflammation and initiated by outside–in signaling, e.g. by activating Toll-like receptors that can convert a wide range of infectious and non-infectious stimuli into NF-κB signaling [1]. Nuclear translocation of NF-κB induces cytokine mRNA transcription, protein translation as well as immediate secretion of the cytokine into the extracellular space [2]. Cytokine receptors detect the cytokine signal and enhance further NF-κB signaling, a process that leads to rapid amplification of local cytokine production and the initiation of tissue inflammation and damage [3]. IL-1β and IL-18 are unique among the proinflammatory cytokines because they do require two signaling steps: first is the nuclear translocation of NF-κB to induce the expression of pro-IL-1β and pro-IL-18 and second is the enzymatic cleavage of immature cytokines into their mature and biologically active forms [4]. The enzymatic cleavage of pro-IL-1β and pro-IL-18 involves the activation of caspase-1 in the intracellular cytosol [4]. The role of caspase-1 for intrarenal IL-1β and IL-18 processing and postischemic renal inflammation was documented a decade ago [5], [6], but the triggers for caspase-1 activation remained enigmatic. The recent discovery of the inflammasomes has provided a novel concept for the enzymatic cleavage of immature cytokines and documented its functional importance for a large number of autoinflamamtory and autoimmune disorders [7]. Inflammasomes are cytosolic molecules that have the capacity to integrate several types of danger signals into caspase-1 activation [7]. The NLRP1 inflammasome is activated by Bacillus anthracis lethal toxin and bacterial peptidoglycans [8], [9]. The NLRC4 inflammasome responds to bacterial flagellin and bacteria containing type III/IV secretion systems like *Salmonella typhimurium and Pseudomonas aeruginosa* [10], [11]. The AIM2 inflammasome detects cytosolic DNA [12]. In contrast to these more or less ligand-specific inflammasomes, the NLRP3 inflammasome and its adaptor molecule ASC are known to convert a broad spectrum of microbial and endogenous triggers into caspase-1 activation [7]. For example, NLRP3 is activated by uric acid crystals, cholesterol crystals, amyloid crystals, as well as high glucose levels which render NLRP3 activation as a crucial element of a number of important inflammatory diseases such as gout, atherosclerosis, amyloidosis and diabetes [13], [14], [15], [16]. Other NLRP3 stimuli include ATP, oxidative stress or biglycan [17], [18], [19], which are all known to contribute to renal cell damage [20], [21]. As such it is not surprising that *Nlrp3*-deficient mice develop less renal IL-1β-dependent inflammation after renal artery clamping [22] or after unilateral ureteral obstruction (UUO) [23]. Given these data we hypothesized a similar role for NLRP3-ASC-caspase-1 for driving IL-1β and IL-18 secretion in the glomerular compartment of the kidney. Some evidence for this concept comes from studies where IL-1R- or IL-18-deficiency was protective in systemic immune responses to foreign antigens that had been implanted into the glomerular basement membrane (GBM), i.e. the autologous version of anti-GBM nephritis [24], [25]. Similar results were obtained by generating Il-18-deficient MRL*lpr* mice with spontaneous immune complex glomerulonephritis [26]. In both of these models, glomerulonephritis develops secondary to systemic immune complex disease, therefore, the role of intrarenal IL-1β and IL-18 production remains unclear. Direct evidence comes from LPS-enhanced heterologous anti-GBM nephritis in rats which were found to be partially protected by anti-IL-1β antibody treatment [27], but a contribution of NLRP3, ASC, and caspase-1 for intrinsic glomerular inflammation is still speculative. We decided to use the passive version of nephrotoxic serum nephritis to induce glomerular inflammation without involving systemic (i.e. adaptive) immune responses. The disease was induced in wildtype mice as well as in mice deficient for IL-1R1, IL-18, NLRP3, ASC, and caspase-1 as an experimental strategy to determine the functional roles of these inflammatory mediators in glomerular inflammation.

## Results

### Heterologous anti-GBM nephritis involves innate but no adaptive immunity

**Figure 1:**
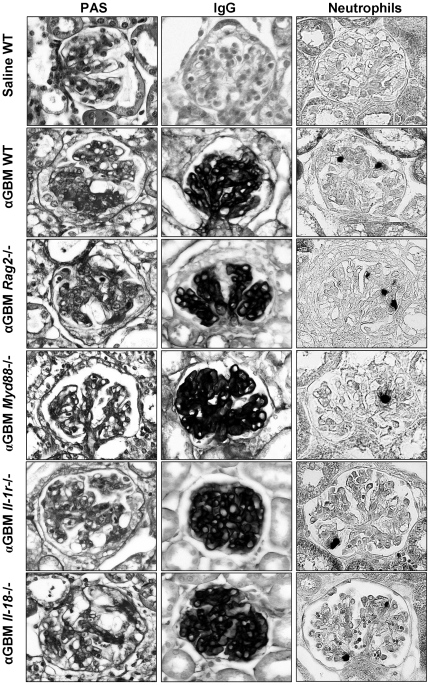
Renal histopathology of heterologous anti-GBM nephritis. Kidneys were obtained from wildtype or gene-deficient C57BL/6 mice on day 7 after saline or antiserum (αGBM) injection. Paraffin-embedded renal sections were stained either with Periodic acid Schiff (PAS) reagent, with anti-IgG or for neutrophils as indicated. The images show representative glomeruli from at least 5 mice in each group at an original magnification 400x.

**Figure 2:**
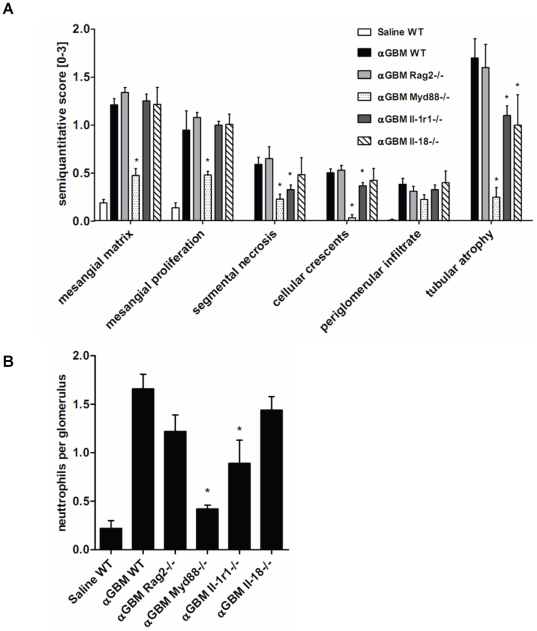
Renal phenotype of heterologous anti-GBM nephritis in *Rag2*-, *Il-1r1*-, *Il-18*- and MyD88 deficient mice. A: Morphometrical assessment of PAS-stained renal sections was performed as described in Methods. WT = wildtype, αGBM = heterologous anti-glomerular basement membrane nephritis. B: Neutrophil counts per glomerulus in mice from all groups. Values represent means±SEM from 5 mice in each group. * p<0.05 versus αGBM WT.

As the GBM antiserum used here was raised in sheep against preparations of rat GBM we first examined its nephritogenic potential in C57BL/6 mice by assessing renal histopathology seven days after a single intravenous injection of antiserum. Immunostaining for sheep IgG revealed a robust positivity exclusively within the glomerular compartment (Figure 1). In contrast staining for mouse IgG was negative excluding autologous *in situ* immune complex formation against sheep IgG (not shown). Glomerular pathology was characterized by an expansion of mesangial matrix, mesangial hypercellularity, focal segmental tuft necrosis, crescent formation (Figure S1), and glomerular neutrophil infiltrates (Figure 1). In contrast, glomerular Mac2+ macrophage or CD3+ T cell infiltrates were not detected (not shown). In the tubulointerstitial compartment focal PAS positive casts were detected in dilated tubular segments as a sign of massive proteinuria and tubular atrophy. Glomerular pathology neutrophil counts were significantly reduced in mice deficient for MyD88, the main signaling adaptor for Toll-like receptors and IL-1R (Figure 1 and 2). By contrast, proteinuria was not much affected by lack of MyD88 indicating that the antiserum has MyD88-independent effects on the glomerular filtration barrier (table 1 and figure S2A). *Rag2*-deficient mice displayed the same phenotype as wildtype mice (Figure 2), indicating that the renal lesions develop independent of T and B cell immunity. Therefore, we considered this protocol of heterologous anti-GBM nephritis to be suitable to study local innate immunity effector pathways in glomerular inflammation and damage.

**Table 1:** Renal function parameters.

	No disease	Anti-GBM GN								
	BL6 WT	BL6 WT	Rag -/-	MyD88-/-	IL1R1-/-	IL18-/-	Nalp3-/-	Asc-/-	NOD/sh	NOD/Casp-1-/-
**Proteinuria**	-	+++	+++	+++	++	++	++	++	+++	+++
**Plasma BUN**	24.60±2.62	25.74±6.75	26.77±4.7	28.95±8.1	29.16±6.13	30.82±6.08	43.76±3.8**	26.85±8.5	29.06±4.29	33.47±5.5

**P<0,01 vs BL6 WT with anti-GBM injection group.

### Glomerular necrosis and crescent formation involve interleukin-1

In order to address a putative role of IL-1 and IL-18 in glomerular inflammation we applied the same disease protocol to C57BL/6 mice deficient either for the IL-1R or for IL-18. An assessment of the aforementioned parameters of glomerular pathology revealed that Il-1r-deficiency partially protected from segmental lesions (65±7% vs. 47±6%) and crescent formation (32±2% vs. 24±2%) as well as from tubular atrophy seven days after antiserum injection (Figures 1 and 2A). By contrast, lack of IL-18 did not significantly protect from glomerular necrosis and crescent formation, however, tubular atrophy was partially reduced as compared to wildtype mice (Figure 1 and 2A). Glomerular neutrophil counts were reduced in IL-1R- but not in IL-18-deficient mice (Figure 2B). By contrast, proteinuria was not much affected by the IL-1R genotype (table 1 and figure S2A). We therefore conclude that IL-1 contributes to glomerular necrosis and crescent formation and that both IL-1 and IL-18 contribute to tubular damage.

### The NLRP3-ASC-caspase-1 axis is induced in anti-GBM disease

**Figure 3:**
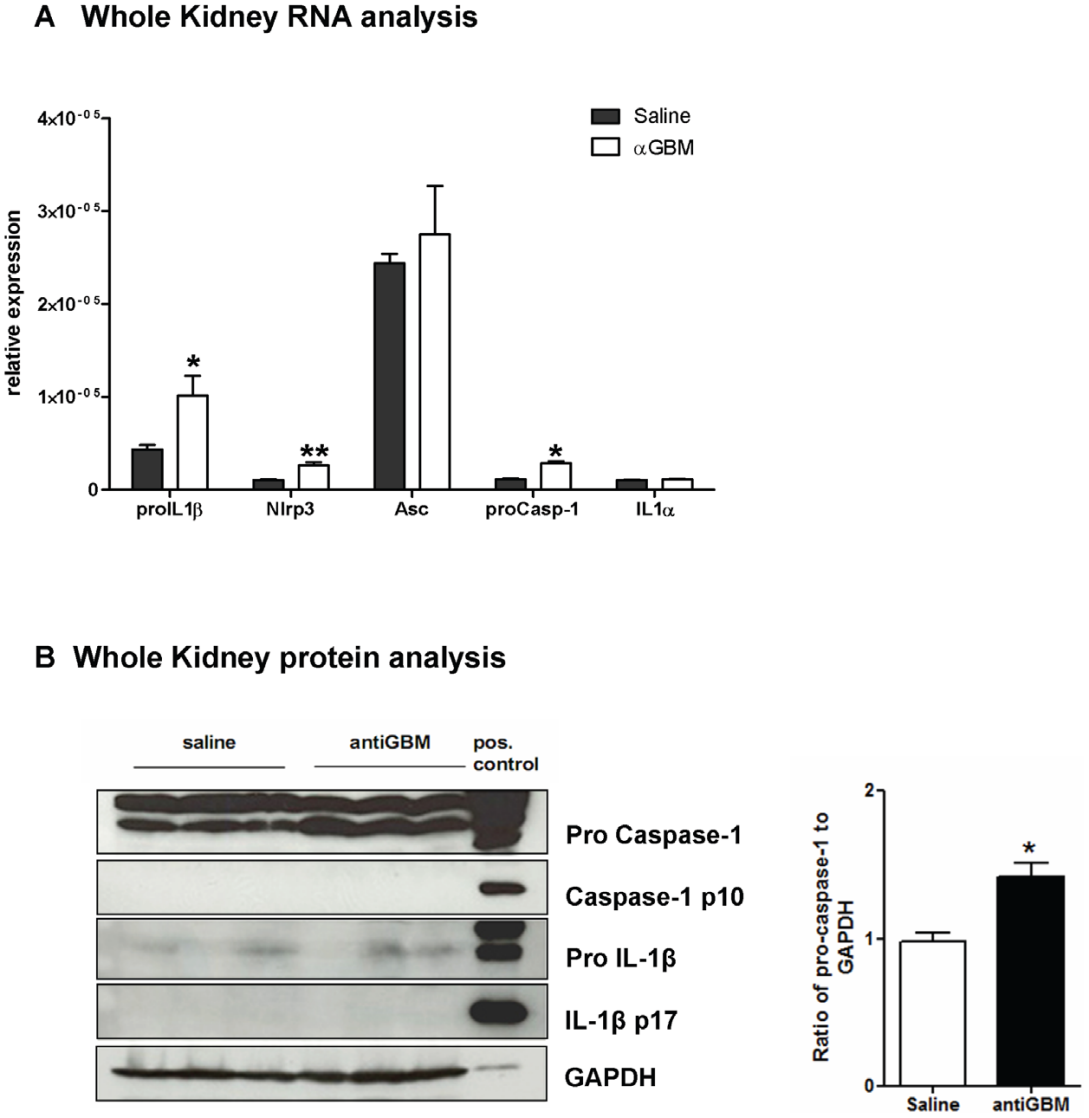
Renal mRNA expression in heterologous anti-GBM nephritis. A: RNA isolates from kidneys of saline- or antiserum-injected C57BL/6 mice underwent quantitative real-time RT-PCR for a number of genes as indicated. Data are expressed as means of the ratio of the specific mRNA versus that of 18S rRNA ± SEM. B: Total kidney protein was isolated from saline and GBM antiserum-injected mice after 7 days. Western blot for caspase-1 illustrates pro caspase-1 at 45 kD and the 10 kD caspase-1 cleaved product which indicates caspase-1 activation. The lower blot shows pro-IL-1β at 31 kD and mature IL-1β at 17 kD. GAPDH was used as a loading control. Note that heterologous anti-GBM glomerulonephritis is not associated with caspase-1 activation and IL-1β release. Stimulated bone marrow dendritic cells (LPS/ATP stimulation) served as a positive control. The increase of pro caspase-1 is quantified by densitometry analysis (ImageJ software).* p<0.05 versus saline control.

The secretion of IL-1β involves inflammasome-dependent activation of caspase-1 [4]. We found pro-IL-1β, caspase-1, NLRP3 mRNA levels were induced in renal samples of C57BL/6 mice injected with the GBM antiserum, albeit at an overall low expression level (Figure 3A). Increase of pro caspase-1 in kidneys of anti-GBM mice could be also detected on protein level (Figure 3B). IL-1α and ASC were expressed in kidneys but were not induced by the GBM antiserum (Figure 3A). The NLRC1, NLRP4 and AIM2 inflammasome mRNA remained undetectable at the mRNA expression level (not shown). The intrarenal expression of NLRP3, ASC, and caspase-1 suggests that they might be involved in the secretion of IL-1β and inflammation during anti-GBM nephritis.

### Anti-GBM disease develops independent of NLRP3, ASC, and caspase-1

**Figure 4:**
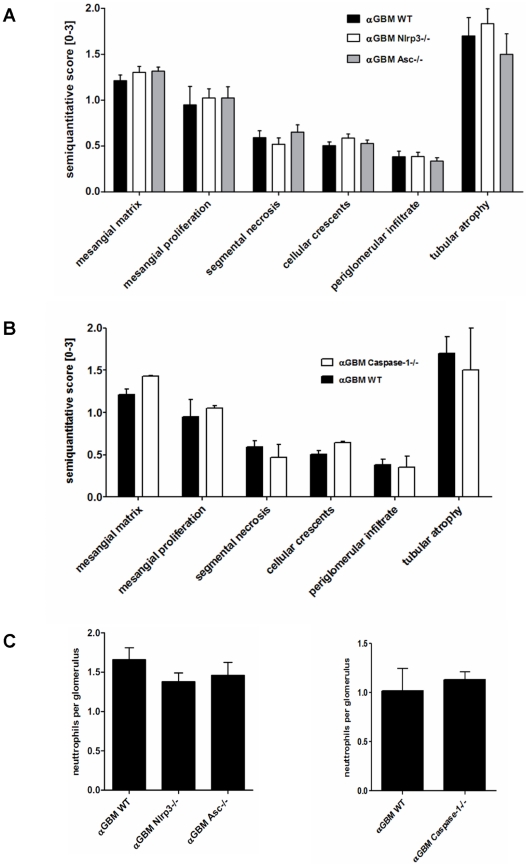
Renal phenotype of anti-GBM nephritis in *Nlrp3*-, *Asc*-, and *caspase-1*- deficient mice. A and B: Morphometrical assessment of PAS-stained renal sections was perfomed as described in Methods. WT = wildtype, αGBM = heterologous anti-glomerular basement membrane nephritis. C and D: Neutrophil counts per glomerulus in mice from all groups. Values represent means±SEM from 5 mice in each group. * p<0.05 versus αGBM WT.

To determine the role of NLRP3, ASC, and caspase-1 in glomerulonephritis, we induced heterologous anti-GBM nephritis in mice with targeted deletions of *Nlrp3, Asc*, and *caspase-1*. When the model was induced following the same protocol as before *Nlrp3*-deficient mice displayed an identical phenotype as compared to wild-type mice in terms of glomerular pathology (Figure 4A) or glomerular neutrophil infitration (Figure 4C). BUN levels were increased in *Nlrp3*-/- mice but this finding did not match with the renal pathology results. Although this findings exclude NLRP3 activation as a mediator of glomerular damage in this model, IL-1β secretion could still be mediated through other inflammasomes that use ASC as a linker for caspase-1 activation [7]. However, ASC-deficiency did not affect glomerular pathology after GBM antiserum injection similar to the phenotype observed in *Nlrp3*-/- mice (Figure 4A and 4C). Next we studied the role of caspase-1 which is the effector molecule in the inflammasome that cleaves pro-IL-1β into its active form. As *spase-1*ca-deficient mice were in a different genetic background respective wildtype control mice were also studied. Again, heterologous anti-GBM nephritis displayed an identical histomorphological and functional phenotype in *caspase-1*-deficient mice as compared to their respective wildtype controls as assessed by scoring glomerular injury (Figure 4B), urinary albumine/creatinine ratio and other renal function parameters (Table 1, figure S2A). This finding was consistent with no detectable active caspase-1 (p10) in kidneys of both untreated and anti-GBM mice as shown by Western blot (Figure 3B). Furthermore, cleavage of pro-IL-1β into active IL-1β (p17) could not at all be detected in kidney isolates, excluding that other proteases induce IL-1β inside the kidney during heterologous anti-GBM nephritis (Figure 3B). We therefore conclude that GBM antiserum induces crescentic glomerulonephritis independent of the NLRP3 inflammasome and of ASC-mediated activation of caspase-1.

### Inflammasome activation in isolated glomeruli

**Figure 5:**
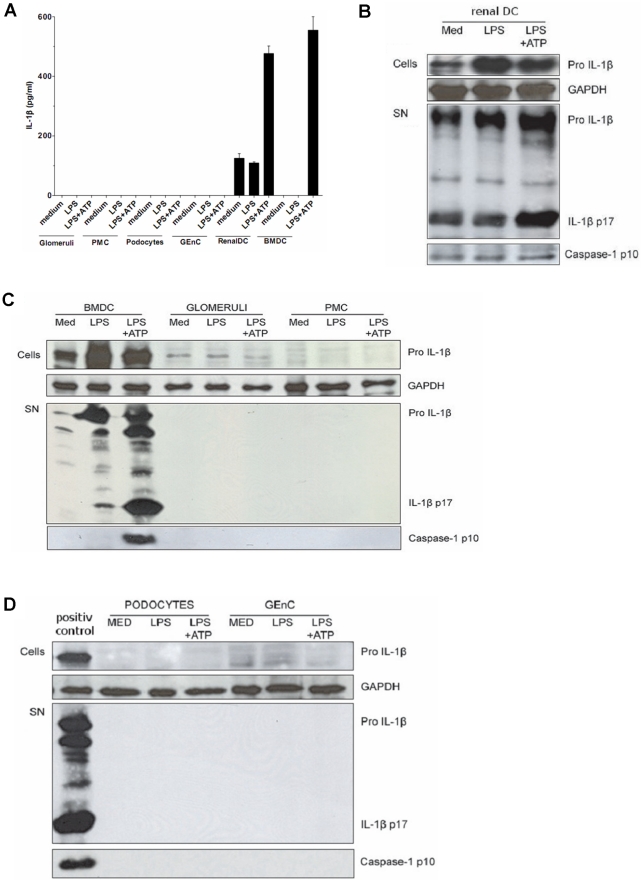
IL-1β release and caspase-1 activation in renal DC, but not in glomeruli and glomerular cells. A. IL-1β release from glomeruli isolated from healthy C57BL/6 wildtype mice, primary mesangial cells (PMC), podocytes, glomerular endothelial cells (GEnC), and renal dendritic cells (DC) stimulated with LPS or LPS followed by ATP was measured by ELISA. Bone marrow dendritic cells (BMDC) served as a positive control. Note that only renal DCs but none of the other glomerular cells released IL-1β after LPS/ATP stimulation. B–D. This was confirmed by Western blot for IL-1β. The blots illustrate the 37 kDa pro-IL-1β in the upper bands and the 17 kDa mature IL-1β in the lower bands. Western blot for caspase-1 illustrates the 10 kDa caspase-1 cleaved product which indicates caspase-1 activation. GAPDH is shown as a loading control. Stimulated bone marrow dendritic cells served as a positive control.

Our finding that the NLRP3 inflammasome does not contribute to glomerular inflammation was unexpected, especially in view of the recently published data on its role in tubulointerstitial injury models [22], [23]. We therefore questioned whether the cells of the glomerular compartment are at all able to activate the NLRP3 inflammasome. To address this question we stimulated freshly isolated glomeruli with LPS or LPS followed by ATP, a potent agonist of the NLRP3 inflammasome, for 6 hours. ATP is released from the mitochondria of necrotic cells and serves as a potent agonist of the NLRP3 inflammasome. In bone marrow dendritic cells LPS stimulation strongly induced pro-IL-1β protein but not caspase-1 activation and, as expected, mature IL-1β secretion into cell culture supernatants depended on additional ATP exposure (Figure 5A, 5C). LPS/ATP stimulation of cultured glomeruli did not result in an increase of pro-IL-1β protein expression or in caspase-1 activation (Figure 5C), suggesting that glomerular cells are unable to secrete mature IL-1β upon ATP exposure.

### Inflammasome activation in glomerular cells

Our *in vivo* data and the lack of IL-1β in glomerular isolates raise doubts about the functional role of the NLRP3 inflammasome-mediated caspase-1 activation in glomerular cells. We therefore examined whether mesangial cells, glomerular endothelial cells (GEnC), podocytes, and renal dendritic cells are able to mount IL-1β release. All glomerular cells highly express TLR2 and TLR4. We prestimulated each of these cell types with TLR4 agonist LPS or TLR2 agonist Pam3CSK4 and challenged them with the NLRP3 agonist ATP as done with isolated intact glomeruli. First, we quantified IL-1β secretion by ELISA in supernatants 24 hours after stimulation. Among all cell types tested only renal dendritic cells induced IL-1β release (Figure 5, figure S2B). Pro-IL-1β protein expression, the mature IL-1β form, and caspase-1 activation were assessed by Western blot after 6 hours of stimulation as before. Consistent with the results from glomerular isolates LPS did not induce pro-IL-1β protein, IL-1β maturation or caspase-1 activation in glomerular cells (Figure 5). Obviously, TLR4 activation does neither induce pro-IL-1β as the necessary first step for inflammasome–mediated IL-1β release nor did it activate caspase-1 in mesangial cells, GEnC, and podocytes. However, renal dendritic cells shared the capacity of caspase-1 activation and IL-1β secretion with BMDCs (Figure 5). We therefore conclude that inside the kidney immune cells like CD11c+ DCs are capable of secreting active IL-1β upon inflammasome activation but this function is not shared by intrinsic glomerular cells due to an inability to induce pro-IL-1β upon TLR4 activation or to activate caspase-1 upon ATP exposure.

### Inflammasome activation in isolated glomeruli from Anti-GBM mouse model

**Figure 6:**
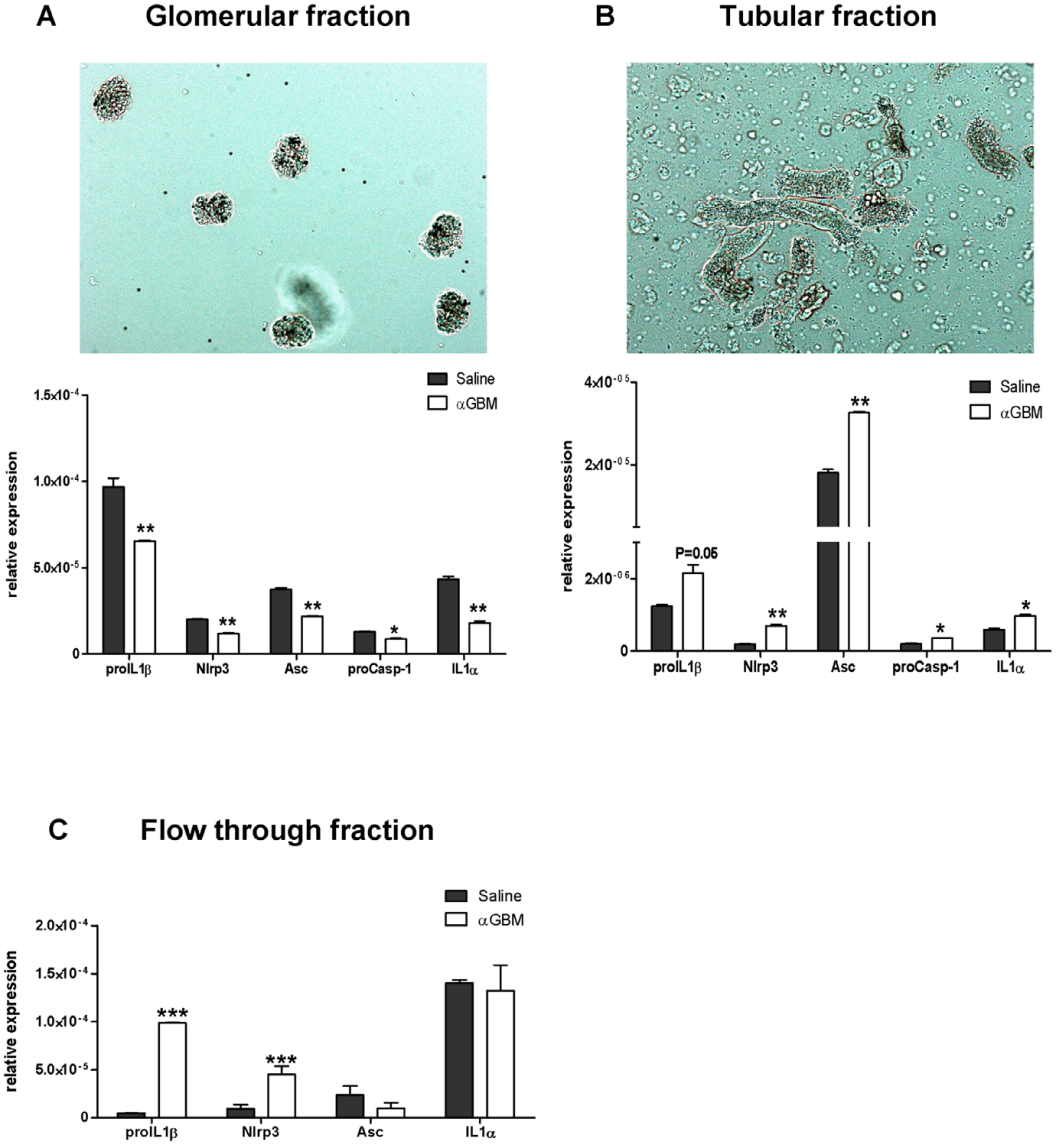
Renal mRNA expression in different compartments of kidney in heterologous anti-GBM nephritis. A. inflammasome related mRNA expression from glomeruli and tubulointerstitial compartments isolated from saline or anti-GBM injected mice on day 7 by using paramagnetic bead isolation technique as mentioned in the methods. mRNA expression levels of proIL1-β, Nlrp3, Asc, proCaspase-1 and IL1-α from glomerular compartment (figure A), Tubular compartment (figure B) and flow through fraction which is devoid of tubules and glomeruli (figure C) are shown. Each analysis represents data from 3 mice in both groups. Microscopic images of glomeruli (figure A) and tubular fraction (figure B) are shown as well. *P<0.05, **P<0.01, ***P<0.001 compared to saline group.

We have shown that glomerular cells are not capable of processing IL1β, but inflammasome associated genes are upregulated in this mouse model (Figure 3). So we determined the specific involvement of glomerular and tubulointerstitial comparments in processing IL1β by isolating glomeruli and tubulointerstitium (Figure 6A,6B) by paramagnetic glomeruli isolation. We did not observe any increase in inflammasome associated genes in glomeruli on day7 (Figure 6A) but tubulointerstitum comparment showed significant upregulation of proIL1β, procaspase-1, Asc and Nlrp3 mRNA levels (Figure 6B,6C).

### Inflammasome expression in human glomerulopathies

**Figure 7:**
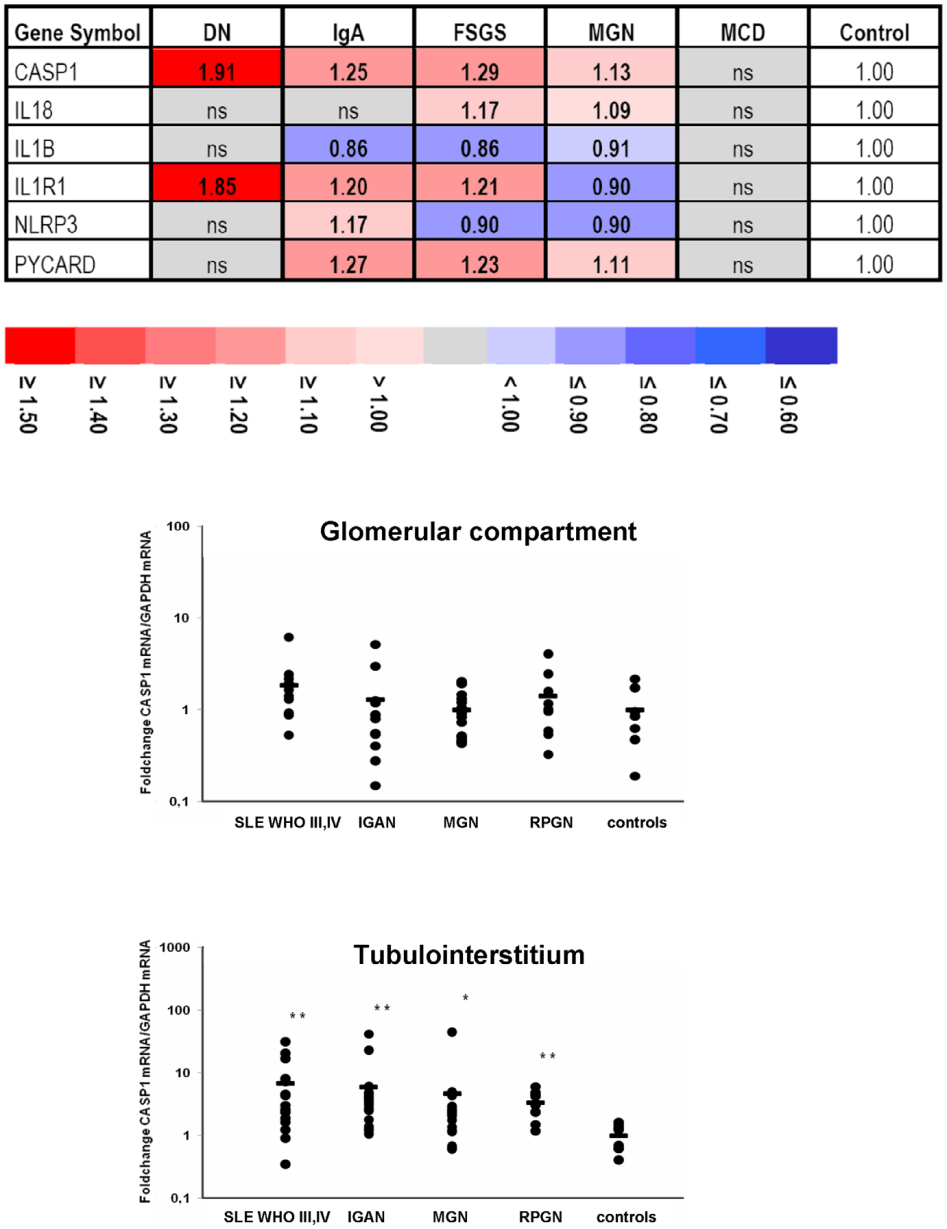
Expression of inflammasome-related genes in human glomerulopathies. A. Gene expression data were obtained from isolated tubulointerstitium of patients with diabetic nephropathy (DN), focal segmental glomerulosclerosis (FSGS), IgA nephropathy (IGAN), non-progressive minimal change disease (MCD), membranous glomerulonephritis (MGN) and controls (pretransplant allograft biopsies) [42], [43]. Gene expression of IL-1 and inflammasome-related genes was significantly regulated in progressive glomerulopathies, but showed no change in minimal change disease compared to controls. Shown are the fold changes of the transcripts with the highest probe coverage. Quantitative CASP1 mRNA expression was validated by real-time RT-PCR on microdissected glomeruli (B) and tubulointerstitial compartments (C) from human biopsies of patients with systemic lupus erythematosus (SLE; n = 16), IgA nephropathy (IGAN; n = 21), membranous nephrophathy (MGN; n = 17), rapidly progressive glomerulonephritis (RPGN; n = 9) and controls (living donors (LD) n = 7, deceased donor (DD) n = 1). CASP1 showed a significant increase in SLE, IgAN and RPGN patients compared to controls. * p<0.05, ** p<0.001 versus controls.

Previously published tubulointerstitial gene expression data from patients with diabetic nephropathy (DN), focal-segmental glomerulosclerosis (FSGS), IgA nephropathy (IGAN), and membranous glomerulonephritis were compared to data of non-progressive proteinuric states such as minimal change disease (MCD), and healthy controls (living allograft donors, LD). Consistent with the finding that progressive proteinuric diseases are associated with tubulointerstitial inflammation, most of the IL-1 and inflammasome related genes were significantly regulated in progressive diseases, whereas transcript levels were unchanged in MCD compared with controls (Figure 7). As CASP1 showed an induction in all progressive diseases, we further dissected its expression in glomerular and tubulointerstitial samples of patients with different progressive glomerulopathies by real-time RT-PCR. Only in the tubulointerstitial compartment of patients with systemic lupus erythematosus (SLE), IGAN, and anti-neutrophil cytoplasmatic antibody (ANCA)-positive, rapidly progressive glomerulonephritis (RPGN) a significant up regulation compared to controls could be observed, while in glomeruli no significant change was seen. Together, genes that are related to the NLRP3-ASC-caspase-1 axis are induced during progressive glomerulonephritis but only in the tubulointerstitium and not in the glomerular compartment of the kidney (Figure 7).

## Discussion

The NLRP3 inflammasome-mediated activation of caspase-1 contributes to a large spectrum of inflammatory diseases but so far little is known about its role in renal inflammation [28]. We had hypothesized that glomerular damage would activate glomerular cells to induce and secrete mature IL-1β and IL-18 by activating the NLRP3-ASC-caspase-1 axis, a hypothesis not supported by our results. In contrary, our data show that intrinsic glomerular inflammation develops independent of the NLRP3-ASC-caspase-1 axis, possibly due to an inability for intrinsic glomerular cells to induce pro-IL-1β and to activate caspase-1 via NLRP3.

The redundant role of NLRP3, ASC, and caspase-1 in antiserum induced glomerular pathology was unexpected because previous studies had documented a non-redundant role of the NLRP3 inflammasome in two models of renal inflammation. Iver, *et al*. reported that *Nlrp3*-deficient mice are partially protected from intrarenal cytokine signaling, neutrophil recruitment, and renal failure associated with postischemic tubular necrosis [22]. Vilaysane, *et al*. induced tubulointerstitial inflammation by UUO in *Nlrp3*-deficient mice and found less tubular damage and interstitial fibrosis as compared to wildtype mice [23]. The latter study addressed the contribution of NLRP3 activation in intrinsic renal cells by experiments with bone marrow chimeric mice and found that NLRP3 is required in both immune cells and non-immune cells for the development of tubular damage and interstitial fibrosis of the same extent as in wildtype mice [23]. Our current study excludes a similar role of NLRP3 in the glomerular compartment. LPS/ATP was unable to elicit caspase-1 activation and IL-1β release in freshly isolated glomeruli, in mesangial cells, glomerular endothelial cells, or podocytes while the same conditions were sufficient to induce IL-1β release in renal dendritic cells. Isolated glomeruli and tubulointerstitial fractions form anti-GBM injected mouse supported our finding that IL-1 beta processing in confined to the extra glomerular compartment. Our data extend on a previous report by Timoshanko, *et al*. that concluded from bone marrow chimera experiments with Il-1β-deficient mice that only leukocyte-derived IL-1β contributes to autologous anti-GBM nephritis [29]. Altogether, these observations have two implications: first, intrinsic glomerular cells cannot secrete IL-1β because they neither induce pro-IL-1β nor do they activate the NLRP3-ASC-caspase-1 axis; second, normal glomeruli harbour negligible numbers of dendritic cells which is consistent with lineage tracking studies of the mouse kidney [30] and with immunohistochemical studies of the human kidney [31]. This conclusion is also supported by our human data showing CASP1 mRNA induction only in the tubulointerstitium, where most of the NLRP3 inflammasome-related genes are found to be induced in human nephropathies and where renal dendritic cells reside [24]. It is intriguing to speculate that the lack of IL-1β secretion by glomerular cells protects the glomerulus from inappropriate inflammation potentially induced by immune complexes, hyperglycemia, oxidative stress, or immunostimulatory crystals.

The rationale for testing the role of NLRP3-ASC-and caspase-1 was based on the phenotype of *Il-1r*- and *Il-18*-deficient mice upon antiserum injection. However, lack of the IL-1R only partially reduced glomerular damage. The IL-1R ligates IL-1α in addition to IL-1β, which may involve an NLRP3- or caspase-1-independent IL-1R agonist that contributes to glomerular pathology and it plays a major role in cell death-induced inflammation [32]. In fact, IL-1α was shown to contribute to the humoral pathways of immune complex glomerulonephritis [24]. IL-18-deficiency had no significant effect on glomerular pathology and only partially reduced tubular atrophy which may relate to a proinflammatory role of IL-18 in this compartment. Our data are in contrast to the documented role of IL-18 signaling in spontaneous lupus-like immune complex glomerulonephritis of MRLlpr(Fas) mice [26]. Still, glomerular inflammation in heterologous anti-GBM disease involves innate rather than adaptive immunity given that the model was MyD88- but not Rag2-dependent. Since injury in this model was previously shown to involve TLR2 and TLR4 [33], [34] we assume that the TLR2/MyD88 and the TLR4/MyD88 pathways predominate for the induction of innate immunity in this model of acute glomerulonephritis.

In summary, heterologous anti-GBM nephritis develops independently of the NLRP3-ASC-caspase-1 axis likely due to an inability for glomerular cells to induce and to secrete IL-1β. Renal dendritic cells secrete IL-1β upon NLRP3 activation mainly in the tubulointerstitial compartment. We therefore conclude that the capacity for triggering innate immunity inside the kidney is compartment-specific. The lack of IL-1β and IL-18 secretion by glomerular cells is another mechanism that prevents inappropriate glomerular inflammation and damage.

## Materials and Methods

### Mice and anti-GBM nephritis model

C57BL/6, NOD/ShiLt, NOD/*Casp1*-/-, Il-1r1-/-, Il-18-/-, *Myd88*-/-, *Rag2*-/- mice were procured from Jackson Laboratories (Bar Habour, MA). *Nlrp3*-/- and *Asc*-/- mice were generously provided by J. Tschopp, Epalinges, Switzerland, and by V. Dixit, Genentech, San Francisco, CA. Mice were housed in groups of 5 mice in filter top cages with unlimited access to food and water. Cages, nest lets, food and water were sterilized by autoclaving before use. All experimental procedures were approved by the local government authorities. 6–8 week old mice were anesthetized using isoflurane for retro-orbital injection of 100 µl of a anti-GBM serum (Sheep anti-Rat glomeruli serum procured from Probetex INC, PTX-001). Urine samples were collected on days 1 and 7 after antiserum injection. On day 7 the mice were sacrificed by cervical dislocation to collect plasma and kidney tissue. Kidneys were kept at −80°C for protein isolation and in RNA later solution at −20°C for RNA isolation. A part of the kidney was also kept in formalin to be embedded in paraffin for histological analysis [35]. Urine samples were then analysed for qualitative estimation of proteinuria by Albustix (Siemens Healthcare diagnostics, Tarrytown, NY, USA) and quantitative estimation of albumin (µg/dl)/ creatinine (mg/dl) ratio using mouse albumin ELISA kit (bethyl laboratories, Montgomery, TX, USA) and creatinine estimation kit (DiaSys diagnostic systems, Holzheim, Germany). Plasma samples were analysed for BUN using urea estimation kit (DiaSys diagnostic systems, Holzheim, Germany). All experiments were approved by the Ethical Committee of the “Regierung von Oberbayern”, Az.: 55.2-1-54-2531-11-10.

### Assessment of renal pathology

Renal sections of 2 µm were stained with periodic acid-Schiff reagent. Glomerular abnormalities were scored in 50 glomeruli per section by a blinded observer. The following criteria were assessed in each of the 50 glomeruli and semiquantitatively scored from 0 = absent, 1 = mild, 2 = moderate, 3 = severe, 4 = extensive, respectively: mesangial matrix expansion, mesangial proliferation, focal necrosis, cellular crescents, periglomerular infiltrate. The extent of tubular atrophy was scored the same way but only one score was given per section. Subcapsular and pole-cutted glomerular cross-sections were ignored. Immunostaining was performed as described using the following primary antibodies: for neutrophils (Serotec, Oxford, UK), mouse IgG and sheep IgG (Caltag Laboratories, Burlingame, CA)[36]. Stained glomerular cells were counted in 15 glomeruli per section. Rat anti-Mouse Ly-6B.2 (clone 7/4, Abd serotec) was used as an antibody to determine neutrophil infiltration.

### RNA preparation and real-time quantitative PCR (RT-PCR)

To determine the activation of inflammasome we analyzed the expression of Nlrp3, IL1β, and caspase-1 by real-time PCR using the following primers:

Nlrp3: AGAAGAGACCACGGCAGAAG (forw), CTTGGACCAGGTTCAGTGT (rev), IL1α: AGCGCTCAAGGAGAAGACC (forw), CCAGAAGAAAATGAGGTCGG (rev), IL1β: TTCCTTGTGCAAGTGTCTGAAG (forw), CACTGTCAAAAGGTGGCATTT (rev), Casp1: TCAGCTCCATCAGCTGAAAC (forw), TGGAAATGTGCCATCTTCTTT (rev), Asc: GAGCAGCTGCAAACGACTAA (forw), GCTGGTCCACAAAGTGTCCT (rev).

Total RNA was isolated from kidneys using Qiagen RNA extraction kit (Düsseldorf, Germany) following the manufacturer's instructions. After quantification RNA quality was assessed using agarose gels. From isolated RNA, cDNA was prepared using reverse transcriptase (Superscript II, Invitrogen, and Carlsbad, CA, USA). Real time PCR was performed using SYBRGreen PCR master mix and was analyzed with a Light Cycler 480 (Roche, Mannheim, Germany). All gene expression values were normalized using 18 s RNA as a house keeping gene. All primers used for amplification were from Metabion (Martinsried, Germany).

### Cell culture studies

Bone marrow derived dendritic cells were generated by established protocols. Glomeruli were isolated from whole kidneys as described elsewhere [37], [38]. Briefly, glomeruli were purified by applying a paramagnetic isolation method following perfusion of mice with magnetic 4.5 µm Dynabeads (Invitrogen). Renal dendritic cells were isolated from whole kidneys by magnetic beads separation (MACS) using CD11c MicroBeads (Miltenyi Biotech, Germany). Purity of CD11c positive renal cells was confirmed by FACS analysis using anti-CD11c antibody (BD Pharmingen, San Diego, CA). Primary mesangial cells were cultured from isolated mouse glomeruli as previously described [39]. GEnC were cultured the same way [40]. Murine podocytes were allowed to proliferate in RPMI 1640 medium (GIBCO/Invitrogen, Paisley, Scotland, UK) containing 10% fetal calf serum, 100 U/ml penicillin, 100 µg/ml streptomycin (PAA Laboratories), and 10 U/ml mouse IFN-γ (ImmunoTools, Firesoythe, Germany) at permissive temperature (33°C), 5% CO_2_. Cells were differentiated at nonpermissive conditions (37°C), 5% CO_2_ without IFN-γ supplement for 10–14 days. RPMI 1640 GlutaMAX™-I medium (Invitrogen) was supplemented with 10% FBS and 1% of penicillin and strepto­mycin (PAA Laboratories, Pasching, Austria). ATP, ultrapure LPS (from Escherichia coli strain K12) and Pam3CSK4 were purchased from Invivogen, (San Diego, CA). All cells were stimulated at a density of 1×10^6^ cells per ml. Cells were stimulated for 3 hours with LPS (50 ng/ml)/ Pam3CSK4 (1 µg/ml), followed by stimulation with ATP (5 mM) for 3 hours. Cell-free supernatants were analyzed for cytokine secretion by ELISA and immunoblot analysis. Cells were lysed for immunoblot analysis or IL-1β by ELISA according to the manufacturer's instructions (BD).

### Immunoblot analysis

To identify the activation of the inflammasome in kidney during heterologus anti-GBM nephritis, we determined the protein content of pro-caspase-1/caspase-1 and pro-IL-1β/IL-1β in total kidney 7 days after serum injection by western blots using antibodies for IL-1β (goat polyclonal anti-IL-1β, R&D Systems, Minneapolis, MN) and caspase-1 (rabbit polyclonal anti-Caspase-1sc-514: Santa Cruz, CA, USA). GAPDH (rabbit polyclonal antibody sc-25778, Santa Cruz Biotechnology) levels were measured as loading control. Densitometry analysis was performed with ImageJ software (NIH).

To evaluate the activation selectively in glomeruli, we isolated protein from glomeruli from saline- and antiserum-injected mice on day 7 (n = 3 in each group). Glomeruli were purified by applying a paramagnetic isolation method following perfusion of mice with magnetic 4.5 µm Dynabeads (Invitrogen) as previously described [37], [38]. Cells were lysed in lysis buffer (1% Nonidet P-40, 50 mM Tris (pH 7.4), 150 mM NaCl) supplemented with a complete protease inhibitor mixture (Roche), and mixed with sample buffer [37], [38]. At the completion of the 6 hour stimulation experiment, supernatants, and cells were collected. Supernatants were concentrated using Amicon Ultra centrifugal filters (Millipore Billerica, MA) and were resolved on 15% SDS Polyacrylamide gels. Cells were washed with PBS and treated with NP-40 lysis buffer (20 mM Tris HCl pH 8, 137 mM NaCl, 10% glycerol, 1% nonidet P-40, 2 mM EDTA and Protease inhibitors). Protein concentration of cell lysates was estimated using Bio-rad protein assay reagent (Bio-rad laboratories GmbH; Munich, Germany). Cell lysates were subjected to SDS-PAGE gel electrophoresis using 15% acrlyamide gels. About 30 µg of protein was loaded per lane and after electrophoresis the proteins were transferred to PVDF membrane. Primary antibodies were goat polyclonal anti-IL-1β (*BAF401: R&D Systems*), rabbit polyclonal anti-Caspase-1(*sc-514: Santa Cruz*), followed by incubation with secondary antibody anti-biotin or anti-rabbit IgG labeled with HRP. GAPDH (rabbit polyclonal antibody sc-25778, Santa Cruz Biotechnology) levels were measured as loading control. Immunostained bands were detected using chemiluminescence kit (ECL kit, GE Healthcare, UK).

### Isolation of different compartmental fractions of renal tissue from saline and anti GBM treated mice

The glomerular fraction was isolated by using magnetic isolation by perfusion of paramanetic beads. The procedure in brief is as follow. Animal was anesthetised by intraperitoneal injection of 2.5% avertin. Animal was then perfused with warm 40 ml PBS containing 8*10^9^ magnetic beads (Dnaybeads, M-450 Epoxy) into left ventricle after lancing the vena cava caudalis. Pressure was maintained at around 60 mm Hg through out the time of perfusion. Successful perfusion will turn the kidney and liver pale. After completion of the perfusion kidneys were harvested and minced into fine pieces. Kidney samples were then digested with collagenase A for 30 minutes at 37°C. Then the digested tissue was passed though 100 µm cell strainer on ice. Then digested and filtered tissue was passed though cell separation magnet (BD IMagnet, BD) and washed for about 5 times to isolate glomeruli fractions. First wash elutes tubulointerstitial part of the kidney. Second wash eluted predominantly the tubular fraction. Remaining fraction was washed carefully until pure fraction of 90% to 95% of glomeruli was obtained upon microscopic observation. All the fractions were then lysed using RNA lysis buffer to prepare mRNA and stored at −20°C until further procedure.

### Human studies

Human renal biopsies from patients and controls were collected within the framework of the European Renal cDNA Bank - Kröner-Fresenius Biopsy Bank [41]. Diagnostic renal biopsies were obtained from patients after informed written consent and with approval of the local ethics committees (Die Spezialisierte Unterkommission- SPUK für Innere Medizin, University of Zurich). Total RNA was isolated from microdissected samples taken from the tubulo-interstitial compartment. The fragmentation, hybridization, staining and imaging was performed according the manufacturer's guidelines (Affymetrix). For a detailed description and access to the deposited raw data of the protocol see reference [42]. A single probe-based analysis tool, ChipInspector (Genomatix Software GmbH, Munich), was used for transcript annotation, total intensity normalization, significance analysis of microarrays and transcript identification based on significantly changed probes [42]. Real-time RT-PCR on biopsies from independent cohorts of patients with systemic lupus erythematosus (SLE; n = 16), IgA nephropathy (IGAN; n = 21), membranous nephrophathy (MGN; n = 17), rapidly progressive glomerulonephritis (RPGN; n = 9) and controls (living donors (LD) n = 7, deceased donor (DD) n = 1) was performed. Reverse transcription and real-time RT-PCR were performed as reported earlier [41]. Pre-developed TaqMan reagents were used for caspase 1 (CASP1, NM_033292.2) and the housekeeper gene GAPDH (Applied Biosystems). Data shown are normalized to GAPDH and target gene expression in the control cohort is set as 1. The mRNA expression was analyzed by standard curve quantification.

### Statistical analysis

Data are presented as mean ± SEM. Comparison of groups was performed using ANOVA and post-hoc Bonferronìs correction was used for multiple comparisons. Human data was analyzed using Kruskall-Wallis and Mann-Whitney U tests (SPSS 17.0, SPSS Inc., Chicago, IL). A value of p<0.05 was considered to indicate statistical significance.

## Supporting Information

Figure S1 **Histopathological assessment of anti-GBM glomerulonephritis.** Kidney tissue was obtained 7 days after antiserum injection, formalin-fixed, and embedded in paraffin. Sections were stained with PAS and the respective morphological characteristics are illustrated by pinpointing those features that were scored for the semiquantitative assessment. Respective scores are given in paracenthesis.
(TIF)

Figure S2 **A. Proteinuria in anti-GBM GN.** Urinary albumin-creatinine ratios as a marker of proteinuria are shown from mice of all genotypes. A logarithmic range was used because all mice with anti-GBM disease developed massive proteinuria in the nephrotic range. **B. No IL-1β release in glomeruli and glomerular cells.** There is no IL-1β release from glomeruli isolated from healthy C57BL/6 wildtype mice, primary mesangial cells (PMC), podocytes, glomerular endothelial cells (GEnC) detectable stimulated with TLR2 agonist Pam3CSK4 or Pam3CSK4 followed by ATP, measured by ELISA. Bone marrow dendritic cells served as a positive control (BMDC).
(TIF)
