# Supplementary figures and images for: Anti-GBM Glomerulonephritis Involves IL-1 but Is Independent of NLRP3/ASC Inflammasome-Mediated Activation of Caspase-1
Source: PLoS One. 2011 Oct 27;6(10):e26778. doi: 10.1371/journal.pone.0026778 (PMC3203143; doi:10.1371/journal.pone.0026778)

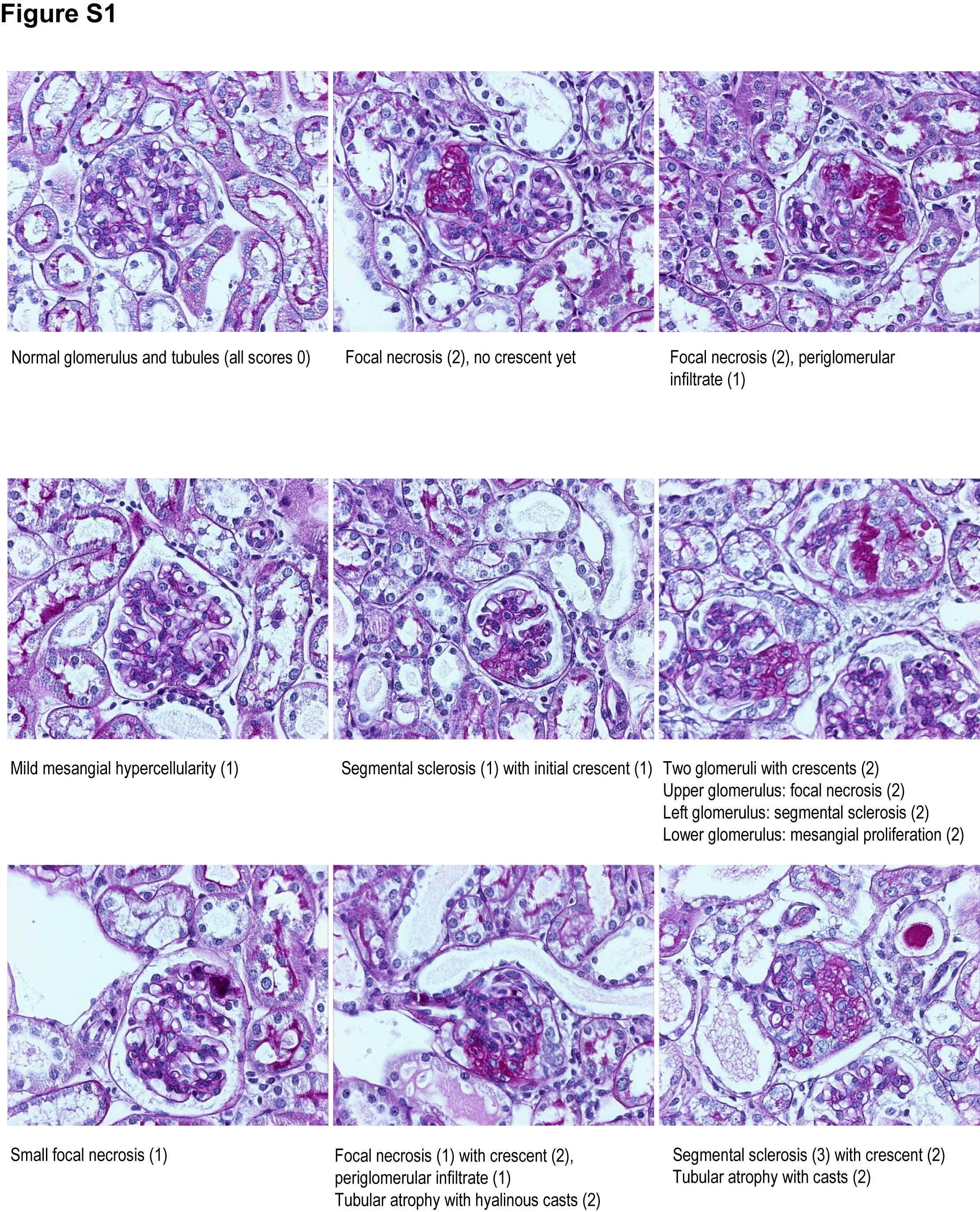

Supplement: Figure S1 — Histopathological assessment of anti-GBM glomerulonephritis. Kidney tissue was obtained 7 days after antiserum injection, formalin-fixed, and embedded in paraffin. Sections were stained with PAS and the respective morphological characteristics are illustrated by pinpointing those features that were scored for the semiquantitative assessment. Respective scores are given in paracenthesis. (TIF) [file pone.0026778.s001.tif]

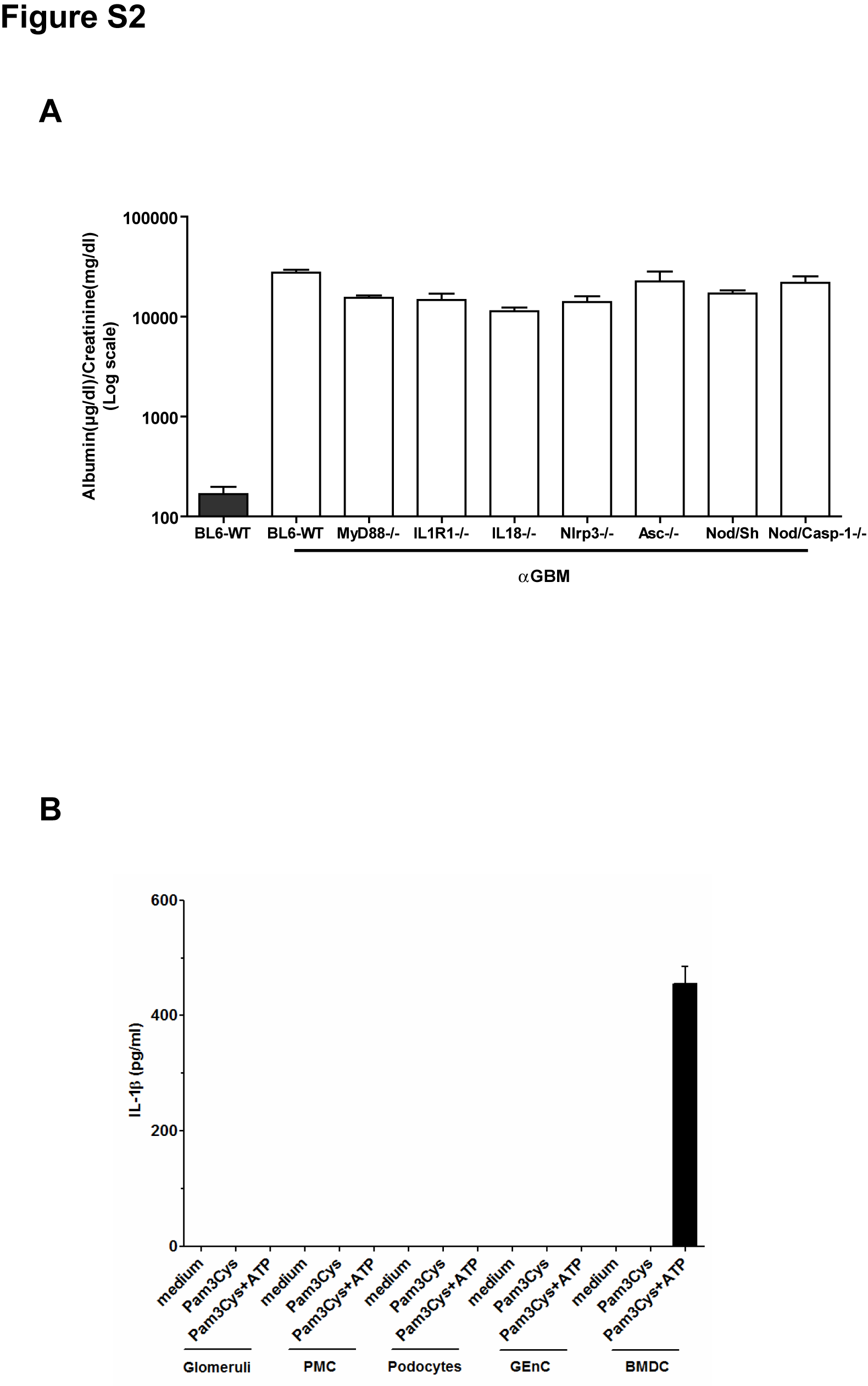

Supplement: Figure S2 — A. Proteinuria in anti-GBM GN. Urinary albumin-creatinine ratios as a marker of proteinuria are shown from mice of all genotypes. A logarithmic range was used because all mice with anti-GBM disease developed massive proteinuria in the nephrotic range. B. No IL-1β release in glomeruli and glomerular cells. There is no IL-1β release from glomeruli isolated from healthy C57BL/6 wildtype mice, primary mesangial cells (PMC), podocytes, glomerular endothelial cells (GEnC) detectable stimulated with TLR2 agonist Pam3CSK4 or Pam3CSK4 followed by ATP, measured by ELISA. Bone marrow dendritic cells served as a positive control (BMDC). (TIF) [file pone.0026778.s002.tif]
